# Dropout and Abstinence Outcomes in a National Text Messaging Smoking Cessation Intervention for Pregnant Women, SmokefreeMOM: Observational Study

**DOI:** 10.2196/14699

**Published:** 2019-10-07

**Authors:** Kristyn Kamke, Emily Grenen, Cendrine Robinson, Sherine El-Toukhy

**Affiliations:** 1 Division of Intramural Research National Institute on Minority Health and Health Disparities National Institutes of Health Bethesda, MD United States; 2 ICF Rockville, MD United States

**Keywords:** smoking cessation, pregnancy, women's health, mHealth, text messaging intervention

## Abstract

**Background:**

Population-level text messaging smoking cessation interventions may reduce racial and ethnic differences in smoking among pregnant women.

**Objective:**

Our objective was to examine racial and ethnic differences in dropout, response, and abstinence rates among users of a US national, publicly available text messaging cessation intervention targeting pregnant women, SmokefreeMOM.

**Methods:**

Participants were online subscribers to SmokefreeMOM who set a prospective quit date within the 9 months before their due date. We examined demographics, smoking frequency, number of cigarettes smoked per day, and prequit time (up to 14 days of preparation time before quit date) as correlates of response rate and abstinence at 8 time points: quit date, day 7, day 14, day 21, day 28, day 35, day 42 (intervention end), and day 72 (1-month follow-up). We conducted survival analysis of time from quit date to dropout by race and ethnicity.

**Results:**

The mean age of the analytic sample of 1288 users was 29.46 (SD 7.11) years. Of these, 65.81% (848/1288) were white, 16.04% (207/1288) were black, 8.86% (114/1288) were Latina, and 9.29% (120/1288) were multiracial, American Indian/Alaska Native, Native Hawaiian Pacific Islander, or other; 82.68% (1065/1288) had some college education or less. Point-prevalence abstinence was 14.51% (157/1082) on quit day, 3.51% (38/1082) at intervention end, and 1.99% (21/1053) at 1-month follow-up. Black users (hazard ratio 0.68, 95% CI 0.51-0.91) and those with a high school degree or less (hazard ratio 0.66, 95% CI 0.49-0.89) or some college education (hazard ratio 0.75, 95% CI 0.57-0.99) were less likely to drop out than whites or users with a bachelor’s degree or higher. Response and abstinence rates were similar across race, ethnicity, and education.

**Conclusions:**

Enrollment was low among racial and ethnic minority women but high among less-educated women. Abstinence at intervention end and 1-month follow-up was lower than that in controlled trials of text messaging cessation interventions for pregnant women (range 7%-20%). Increasing the reach, engagement, and effectiveness of SmokefreeMOM, especially among women with high rates of smoking during pregnancy, must be prioritized.

## Introduction

### Background

Pregnant women are a priority population for smoking cessation efforts [1,2]. Smoking during pregnancy confers negative health outcomes on the mother, fetus, and infant, including preterm birth, low birthweight, birth defects, and infant death [1,3]. Nevertheless, 6.9% (264,920) of pregnant women in the United States reported smoking in 2017, of whom 79% continued to smoke throughout pregnancy [4]. Further, evidence suggests that 31% to 52% of women will resume smoking postnatally [5], exposing their babies to secondhand smoke-related problems [6].

Racial and ethnic disparities in smoking during pregnancy are apparent. American Indian/Alaska Native (AI/AN) (16.4%) and white (10.1%) women have the highest smoking rates, exceeding the US national average of smoking during pregnancy [4]. Black and Native Hawaiian and Pacific Islander (NHPI) women have the next highest rates (5.6% and 4.6%, respectively), followed by Latina and Asian women (1.8% and 0.5%, respectively) [4]. Importantly, pregnant women, especially racial and ethnic minorities, may underreport their smoking status. Biological measures show that nicotine exposure among black and Latina pregnant women is 2 to 4 times higher than self-reported rates [7]. Whereas rates of smoking during pregnancy in the United States decreased 4% to 5% annually among white and Latina women from 1985 to 2013, rates have not decreased among black women over time [8]. Additionally, smoking cessation rates increased 9% annually from 1985 to 2013 among Latina women but not among white or black women [8].

Other risk factors can exacerbate the adverse health outcomes of smoking during pregnancy among minorities. Greater psychosocial stress and experiences of discrimination among racial and ethnic minority women heighten the risk of smoking during pregnancy and negatively affect prenatal and postnatal health [9,10]. Indeed, compared with white women, black, Latina, AI/AN, and NHPI women experience higher rates of smoking-related negative birth outcomes, including preterm birth, lower birthweight, and infant death [4,11]. Compared with white women, black, Latina, AI/AN, and NHPI women are less likely to begin prenatal care in the first trimester [4,11], limiting time for health practitioners to intervene in their smoking behavior, and are less likely to breastfeed [4,12], a key protective factor against smoking during and after pregnancy [13].

Behavioral interventions are necessary cessation aids for pregnant women, as the effectiveness and safety of nicotine replacement therapy during pregnancy is not well established [1,14]. Population-level short message service (SMS) text messaging interventions have been used widely and effectively for smoking cessation [15,16]. They can be tailored to deliver pregnancy-focused information and have high penetration, allowing vulnerable groups to access cessation services typically unavailable to them due to financial or logistical constraints [17,18]. These interventions also reduce pregnancy-specific barriers to seeking smoking cessation resources, such as fear of stigmatization or legal repercussions [17,18].

SMS text messaging smoking cessation interventions are efficacious among the general population of smokers [15,16]. However, few SMS text messaging interventions have been developed for and evaluated among pregnant women [17]. One available intervention is SmokefreeMOM, an evidence-informed SMS text messaging smoking cessation intervention for pregnant women. SmokefreeMOM is offered within a suite of Web- and mobile-based smoking cessation resources, accessible through Smokefree.gov, a US National Cancer Institute initiative [19].

### Objective

Evidence shows that SmokefreeMOM is acceptable and engaging, improves motivation for quitting, reduces craving symptoms, and promotes smoking abstinence [20,21]. However, to our knowledge, no study has evaluated the population-level implementation of SmokefreeMOM. Of interest is whether SmokefreeMOM yields comparable retention, response, and abstinence rates among racial and ethnic subgroups of pregnant smokers. Thus, this study examined racial and ethnic differences in dropout, response, and abstinence rates among SmokefreeMOM users.

## Methods

### Intervention Description

SmokefreeMOM is a free, publicly available smoking cessation intervention, accessible to anyone in the United States with an SMS text messaging–enabled mobile phone. It was developed with input from pregnant smokers and is grounded in social cognitive theory and other proven behavioral strategies for smoking cessation [22-24]. Women sign up for SmokefreeMOM online at Smokefree.gov or by texting the keyword “Mom” to 222888. Upon enrollment, users select their goal for the program: “I want to quit smoking” or “I am not ready to make a change but would like to receive messages on smoking and health.” A previous iteration also allowed users to select “I want to cut back.” Users who are not ready to quit or want to cut back receive an alternative message library. Users who want to quit smoking set a quit date recommended to be within 14 days of sign-up and answer demographic and smoking-related questions. Women can reset their quit date and restart the program by texting “DATE” at any time. Prior to the quit date (ie, prequit period), users receive up to 34 messages over a maximum of 14 days to prepare them to quit smoking. The self-set quit date triggers the 42-day SmokefreeMOM intervention with 101 smoking cessation messages, including behavioral challenges, facts on the effects of smoking on a baby’s development, advice from former pregnant smokers, and links to smoking cessation resources. Users receive up to 313 days of messages related to maternal and child health that correspond with their due date, continuing beyond the 42-day intervention.

### Study Population

In 2764 user records available from April 2014 to June 2018, 584 users were not asked the race, ethnicity, and education questions because they enrolled via mobile keyword. This reduced our initial sample to 2180. We devised our exclusion criteria around SmokefreeMOM parameters (Table 1). First, for users who reset their quit date, we used data from their most recent quit date and excluded prior records for each user. Second, we excluded users who were not ready or did not want to quit smoking, had no quit date or due date, retrospectively set a quit date, reported a due date before their quit date, or reported a due date that was more than 280 days after their sign-up date (ie, time from sign-up to due date), which is the maximum gestational period [25]. We also excluded users who dropped out of the study prior to the prequit period without receiving any intervention dose. Finally, we downloaded data on June 30, 2018, which we deemed to be the end-of-study date. Accordingly, we excluded users whose quit date was less than 42 days prior to the end-of-study date because they did not have the opportunity to complete the intervention. The final analytic sample comprised 1288 users.

**Table 1:** Exclusion criteria (N=2180 Web-enrolled users in SmokefreeMOM between April 2014 and June 2018).

First reason for exclusion	N (%)^a^
Multiple sign-ups	458 (21.01)
Not ready to quit	146 (6.70)
No quit date	28 (1.28)
Quit date precedes sign-up date	60 (2.75)
Due date is more than 280 days after sign-up date	32 (1.47)
No due date	1 (0.05)
Due date precedes quit date	101 (4.63)
Opted out before prequit period	12 (0.55)
Quit date less than 42 days before end of study^b^	54 (2.48)
Total excluded from study	892 (40.92)
Total included in study	1288 (59.08)

^a^N (%) for multiple sign-ups refers to records rather than unique participants; number of records per user ranged from 2 to 37 (mean 2.92, SD 3.08); n (%) for all other exclusion criteria represents unique users excluded.

^b^End-of-study date was June 30, 2018, when the data were pulled.

### Measures

At sign-up, users reported their age, race, ethnicity, educational attainment, smoking frequency, cigarettes smoked per day, zip code, and whether they used a Web-enabled phone. We combined race and ethnicity, in which we classified ethnically Latina women as such regardless of their race and classified all others by their race (eg, black). Users reported their due date and selected a quit date (if their goal was to quit smoking). Three dates were captured automatically: (1) sign-up, (2) opt-out if user texted “STOP” or if text messages were undeliverable to the registered phone number, and (3) quit date reset, if any. We used self-reported and automatically captured dates to derive the following time variables: (1) time from sign-up to quit date, (2) prequit time, and (3) time from quit date to dropout.

We used time from sign-up to quit date to derive a continuous prequit-time variable, which we used as a covariate in our analyses because of associations between preparation stage and intervention dose with smoking cessation [16,26]. Although users were prompted to set a quit date within 14 days of sign-up, some users selected a quit date beyond the maximum recommended prequit period, during which they received no intervention messages. For these users, we reset their prequit time to 14 days. We made no changes for those who did not exceed 14 days. For example, if a woman set a quit date 30 days after sign-up, we reset her prequit time to 14 days; however, if a woman set her quit date at 7 days after sign-up, her prequit time remained at 7 days. Time from quit date to dropout ranged from –14 (for women who dropped out 14 days before their quit date on the first day that prequit intervention content was delivered) to 42 (for women who dropped out on the last day of the intervention).

We derived a binary dropout variable from the time from quit date to dropout variable, in which we considered users to be *noncompleters* if they texted “STOP” to opt out or for whom text messages were undeliverable by the intervention between day –14 and intervention end (day 42), or to be *completers* if they were retained beyond the intervention end, regardless of their engagement with the intervention. SmokefreeMOM prompted users via text message to report their smoking status every week starting on the quit date and ending on intervention end day. They also reported their smoking status at 1, 3, and 6 months after intervention end (ie, days 72, 132, and 222). Smoking status was captured by a yes/no question: “Hi there! Have you smoked in the last 7 days?” We derived a binary responding status variable, in which we deemed users to be *responders* if they responded to the smoking status prompt with yes or no and as *nonresponders* if they did not respond.

### Data Analysis

Almost one-third of users (382/1288, 29.66%) had missing values on 1 or more user characteristics, suggesting listwise deletion would bias results [27]. The Little chi-square test [28] showed that data were not missing completely at random (χ^2^_81_=117.8, *P*=.01). Statistical analyses are likely to be biased when more than 10% of data are missing [29,30]. Since multiple imputation methods are preferred when data are not missing completely at random [30], we imputed missing data (n=20) for race, ethnicity, educational attainment, smoking frequency, cigarettes per day, and region with input data that met our inclusion criteria. Because we imputed categorical variables, we specified a logistic prediction model and used a generalized logit model for nonordinal variables (ie, race, region). Although we have a combined race and ethnicity variable, we imputed race and ethnicity separately because some users were missing only 1 of the 2 variables. Age, ownership of a Web-enabled phone, and prequit time had no missing data points and were used as covariates in multiple imputations models. We winsorized self-reported age that fell outside 10 to 54 years (17/1288, range 55-99) [31] to preserve data from outliers while minimizing their impact on the results [32]. This age range is consistent with the US Centers for Disease Control and Prevention’s range of probable ages for becoming pregnant [31].

Using imputed data, we conducted a Cox regression survival analysis of time from quit date to dropout by race and ethnicity. We did not detect violations of multicollinearity or proportionality of hazards assumptions. Users were right-censored at 42 days after quit date. We conduced logistic regressions to examine correlates of response and abstinence rates on quit date through 1-month follow-up. We limited response rate models to users who remained in the intervention and had the opportunity to respond to each smoking status prompt; thus, the n for response rate models varied at each assessment time. We based abstinence models on an intent-to-treat approach in which we considered users who did not respond to the smoking status prompt or had dropped out before the date of prompt to be smokers. Cox regression survival analysis and abstinence models included users who did not drop out prior to the quit date and had the opportunity to respond to the first smoking status prompt on the quit date (n=1082). We adjusted dropout, response rate, and abstinence models for age, race and ethnicity, educational attainment, region, smoking frequency, cigarettes smoked per day, and prequit time.

## Results

### Participant Characteristics

Among the analytic sample, 65.81% (848/1288) were white, 16.04% (207/1288) were black, 8.86% (114/1288) were Latina, and 9.29% (120/1288) were multiracial, Asian, AI/AN, NHPI, or other race (Tables 2 and 3; Multimedia Appendix 1 shows complete case user characteristics). Approximately 17.32% (223/1288) had a bachelor’s degree or higher, whereas 82.68% (1065/1288) had some college education, or a high school degree or less. On average, users were 29.46 years old and signed up for SmokefreeMOM at the beginning of their second trimester (ie, 3.6 months pregnant) and had around 5.4 months until their due date.

### Dropout Rates

**Figure 1:**
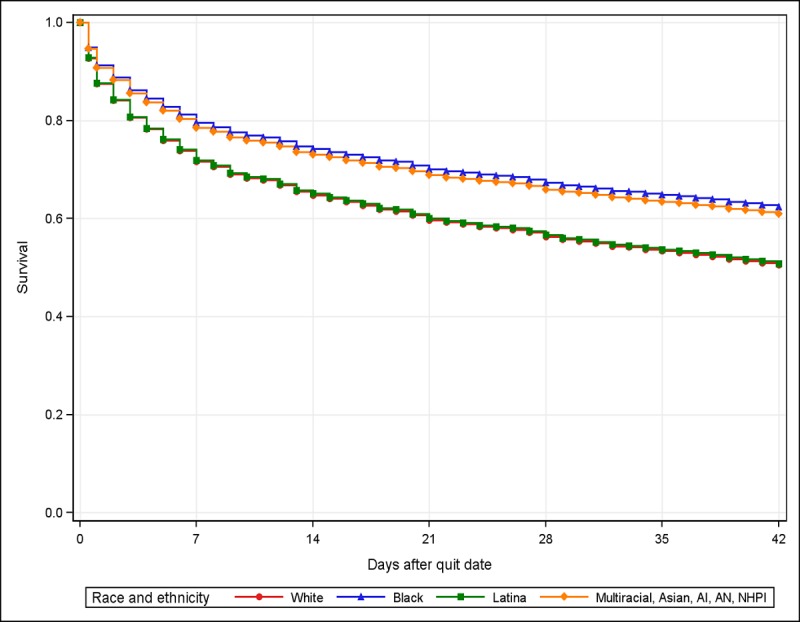
Survival analysis of days in SmokefreeMOM by race and ethnicity adjusted for age (winsorized), educational attainment, region, smoking frequency, cigarettes per day, and prequit time, imputed data (N=1082). AI/AN: American Indian/Alaska Native; NHPI: Native Hawaiian and Pacific Islander.

Of all SmokefreeMOM users, 15.99% (206/1288) dropped out before their quit date and 39.52% (509/1288) dropped out on or after their quit date. Compared with white users who remained in the program until their quit date, black users were less likely to drop out of SmokefreeMOM before intervention end (hazard ratio [HR] 0.68, 95% CI 0.51-0.91, Figure 1, Table 4). At the mean of the covariates, the survival rate was around 80% for black women on day 7 and around 62% on day 42 (Figure 1). Women with some college education (HR 0.75, 95% CI 0.57-0.99) and those with high school education or less (HR 0.66, 95% CI 0.49-0.89) were less likely to drop out of SmokefreeMOM than were women with bachelor’s degrees or higher. A longer prequit time (ie, 0-14 days of preparation time before quit date) was associated with a lower likelihood of dropping out (HR 0.97, 95% CI 0.96-0.99). A logistic regression analysis for dropout that included women who dropped out before and on or after the quit day showed similar results (Multimedia Appendix 2).

**Table 2:** SmokefreeMOM user characteristics, imputed data (N=1288).

Characteristics		Total	Noncompleters^a^	Completers^b^
Users, n (%)		1288 (100.00)^c^	715 (55.51)^c^	573 (44.49)^c^
**Age, years**				
	Mean (SD)	29.58 (7.64)	29.64 (7.68)	29.49 (7.60)
	5% trimmed mean (SD)	29.02 (6.35)^d^	29.09 (6.33)^e^	28.93 (6.38)^f^
	Range	14-99	16-99	14-72
	Median (IQR^g^)	29.00 (10.00)	29.00 (10.00)	28.00 (10.00)
**Age (winsorized), years**				
	Mean (SD)	29.46 (7.11)	29.52 (7.05)	29.39 (7.19)
	5% trimmed mean (SD)	29.02 (6.35)^d^	29.09 (6.33)^e^	28.93 (6.38)^f^
	Range	14-54	16-54	14-54
	Median (IQR)	29.00 (10.00)	29.00 (10.00)	28.00 (10.00)
**Time from sign-up date to due date, days**				
	Mean (SD)	161.21 (73.65)	164.73 (75.57)	156.82 (71.01)
	5% trimmed mean (SD)	164.49 (75.93)^d^	168.45 (78.25)^e^	159.47 (72.34)^f^
	Range	0-279	0-279	0-279
	Median (IQR)	175.50 (117.00)	184.00 (113.00)	167.00 (113.00)
**Time from sign-up to quit date, days**				
	Mean (SD)	10.41 (15.74)	9.38 (14.06)	11.70 (17.53)
	5% trimmed mean (SD)	8.04 (9.60)^d^	7.58 (7.65)^e^	8.93 (12.65)^f^
	Range	0-197	0-197	0-144
	Median (IQR)	7.00 (13.00)	7.00 (13.00)	7.00 (13.00)
**Prequit time, days**				
	Mean (SD)	6.12 (5.53)	5.20 (5.29)	7.28 (5.61)
	5% trimmed mean (SD)	6.03 (5.83)^d^	5.00 (5.58)^e^	7.31 (5.91)^f^
	Range	0-14	0-14	0-14
	Median (IQR)	5.00 (12.00)	3.00 (10.00)	7.00 (13.00)
**Time from quit day to dropout, days^h^**				
	Mean (SD)	10.70 (11.74)^i^	10.70 (11.74)^i^	N/A^j^
	5% trimmed mean (SD)	9.67 (11.92)^k^	9.67 (11.92)^k^	N/A
	Range	0-42^i^	0-42^i^	N/A
	Median (IQR)	6.00 (16.00)^i^	6.00 (16.00)^i^	N/A

^a^Noncompleters were users who opted out of the intervention any time on or between day –14 (14 days before quit date) and intervention end (day 42).

^b^Completers were users who remained in SmokefreeMOM until after intervention end.

^c^Imputed Ns have 20 records and thus n is 1/20th of a subject rounded to the nearest integer.

^d^N=1158.

^e^n=643.

^f^n=515.

^g^IQR: interquartile range.

^h^Among those who made it to their quit date.

^i^N=509.

^j^Not applicable.

^k^N=457.

**Table 3:** SmokefreeMOM user characteristics, imputed data (N=1288).

Characteristics		Total, n (%)^a^	Noncompleters^b^, n (%)^a^	Completers^c^, n (%)^a^
**Race and ethnicity**				
	White	848 (65.81)	499 (69.80)	349 (60.84)
	Black	207 (16.04)	88 (12.24)	119 (20.78)
	Latina	114 (8.86)	69 (9.62)	45 (7.91)
	Multiracial, Asian, AI/AN^d^, NHPI^e^, other^f^	120 (9.29)	60 (8.34)	60 (10.48)
**Education**				
	High school or less	513 (39.86)	267 (37.32)	247 (43.02)
	Some college	552 (42.82)	302 (42.17)	250 (43.63)
	College graduate or higher	223 (17.32)	147 (20.50)	77 (13.35)
**Region^g^**				
	Northeast	180 (13.94)	105 (14.69)	75 (13.00)
	Midwest	332 (25.74)	201 (28.06)	131 (22.84)
	South	569 (44.15)	293 (41.01)	275 (48.07)
	West	208 (16.17)	116 (16.24)	92 (16.08)
**Smoking frequency**				
	Nondaily	143 (11.09)	67 (9.39)	76 (13.21)
	Daily	1145 (88.91)	648 (90.61)	497 (86.79)
**Cigarettes per day**				
	Light (<10 cigarettes)	762 (59.12)	397 (55.57)	364 (63.55)
	Moderate (11-19 cigarettes)	416 (32.29)	240 (33.60)	176 (30.65)
	Heavy (≥20 cigarettes)	111 (8.59)	77 (10.83)	33 (5.79)
**Web-enabled phone**				
	Yes	1238 (96.12)	687 (96.08)	551 (96.16)
	No	50 (3.88)	28 (3.92)	22 (3.84)
**Time of dropout^h^**				
	Prior to quit date	206 (15.99)	206 (15.99)	N/A^i^
	On or after quit date	509 (39.52)	509 (39.52)	N/A

^a^Imputed Ns have 20 records and thus n is 1/20th of a subject rounded to the nearest integer.

^b^Noncompleters were users who opted out of the intervention any time on or between day –14 (14 days before quit date) and intervention end (day 42).

^c^Completers were users who remained in SmokefreeMOM until after intervention end.

^d^AI/AN: American Indian/Alaska Native.

^e^NHPI: Native Hawaiian and Pacific Islander.

^f^Complete case sample was 4.72% (58/1230) multiracial, 1.14% (14/1230) Asian, 0.73% (9/1230) AI/AN, 0.49% (6/1230) NHPI, and 2.11% (26/1230) other.

^g^Users provided their zip codes, which were automatically converted into US state. We categorized states into US Census Bureau region. We categorized 1 user who lived in Puerto Rico, for which there is no census region, into South to retain her data in the analyses.

^h^The remaining 44.49% (573/1288) did not drop out prior to intervention end.

^i^Not applicable.

**Table 4:** Hazard ratios of dropping out by user characteristics, imputed data (N=1082).

Characteristics		Hazard ratio	95% CI	*P* value
Age (winsorized)		0.99	0.97-1.00	.11
**Race and ethnicity (reference: white)**				
	Black	0.68	0.51-0.91	.009
	Latina	0.99	0.72-1.37	.95
	Multiracial, Asian, AI/AN^a^, NHPI^b^, other	0.72	0.51-1.01	.06
**Education (reference: college graduate or higher)**				
	High school or less	0.66	0.49-0.89	.006
	Some college	0.75	0.57-0.99	.04
**Region (reference: South)**				
	Northeast	1.15	0.87-1.51	.32
	Midwest	1.20	0.96-1.49	.10
	West	1.11	0.85-1.44	.44
**Cigarettes per day (reference: light)**				
	Moderate	1.03	0.85-1.26	.74
	Heavy	1.38	0.99-1.92	.06
**Smoking frequency (reference: nondaily)**				
	Daily	1.32	0.96-1.80	.08
Prequit time		0.97	0.96-0.99	.001

^a^AI/AN: American Indian/Alaska Native.

^b^NHPI: Native Hawaiian and Pacific Islander.

### Response and Abstinence Rates

Response rates averaged 29.48% (319/1082) on quit date, were between 10.23% (62/606) and 18.45% (150/813) through day 35, then dropped below 9.86% (57/578) for intervention end and 1-month follow-up. Overall abstinence ranged from 14.51% (157/1082) on quit day to 3.51% (38/1082) at intervention end and 1.99% (21/1053) at 1-month follow-up (Multimedia Appendix 3). Although no clear pattern of associations emerged between user characteristics and either response rate or abstinence (Tables 5-8), some significant associations are noteworthy. Black users (adjusted odds ratio [aOR] 0.36, 95% CI 0.15-0.91), those with high school education or less (aOR 0.29, 95% CI 0.15-0.56), and daily smokers (aOR 0.54, 95% CI 0.31-0.94) were less likely to respond at individual time points throughout the intervention than were their respective reference groups. Similarly, users with high school education or less (vs those with bachelor’s degrees or higher; aOR 0.39, 95% CI 0.17-0.92) were less likely to be abstinent on day 7, moderate smokers (vs light smokers) were more likely to be abstinent on day 35 (aOR 2.31, 95% CI 1.12-4.75), and daily smokers (vs nondaily) were less likely to be abstinent on quit day (aOR 0.42, 95% CI 0.26-0.67), day 14 (aOR 0.38, 95% CI 0.20-0.74), and 1-month follow-up (aOR 0.27, 95% CI 0.08-0.89). Longer prequit intervention time was associated with a reduced likelihood of abstinence on quit day (aOR 0.96, 95% CI 0.93-0.99). Sensitivity analyses using unimputed data with listwise deletion showed fairly comparable results (Multimedia Appendix 4).

**Table 5:** Associations between user characteristics and response rates: from quit date to days 7-21, imputed data.

Characteristics		Quit date (n=1082)^a^		Day 7 (n=813)^a^		Day 14 (n=727)^a^		Day 21 (n=678)^a^	
		aOR^b^ (95% CI)	*P* value	aOR (95% CI)	*P* value	aOR (95% CI)	*P* value	aOR (95% CI)	*P* value
Age (winsorized)		0.99 (0.97-1.01)	.27	0.98 (0.95-1.01)	.25	0.99 (0.97-1.03)	.73	1.00 (0.97-1.04)	.80
**Race and ethnicity (reference: white)**									
	Black	1.05 (0.71-1.54)	.82	0.85 (0.49-1.45)	.55	0.87 (0.50-1.53)	.64	1.01 (0.57-1.78)	.97
	Latina	0.58 (0.33-1.01)	.05	0.63 (0.28-1.41)	.26	1.22 (0.56-2.62)	.62	0.35 (0.10-1.19)	.09
	Multiracial, Asian, AI/AN^c^, NHPI^d^, other	0.86 (0.53-1.39)	.53	0.86 (0.47-1.60)	.64	1.61 (0.87-2.97)	.13	1.04 (0.49-2.22)	.91
**Education (reference: college degree or higher)**									
	High school or less	0.64 (0.40-1.02)	.06	0.29 (0.15-0.56)	<.001	0.56 (0.26-1.20)	.14	0.73 (0.30-1.75)	.47
	Some college	0.98 (0.65-1.48)	.93	0.64 (0.38-1.06)	.08	1.10 (0.57-2.14)	.77	1.45 (0.65-3.22)	.36
**Region (reference: South)**									
	Northeast	1.20 (0.80-1.82)	.38	1.25 (0.71-2.17)	.44	1.21 (0.68-2.16)	.52	1.12 (0.58-2.15)	.74
	Midwest	1.13 (0.81-1.58)	.47	0.96 (0.59-1.53)	.85	1.41 (0.88-2.28)	.16	1.44 (0.87-2.38)	.15
	West	1.26 (0.85-1.85)	.25	1.32 (0.78-2.23)	.30	1.02 (0.56-1.85)	.94	0.48 (0.21-1.06)	.07
**Cigarettes per day (reference: light)**									
	Moderate	1.21 (0.89-1.65)	.22	1.18 (0.78-1.78)	.45	1.49 (0.96-2.32)	.07	0.91 (0.55-1.50)	.71
	Heavy	0.92 (0.52-1.63)	.78	0.64 (0.24-1.74)	.38	1.13 (0.46-2.74)	.79	1.58 (0.65-3.83)	.32
**Smoking frequency (reference: nondaily)**									
	Daily	0.69 (0.46-1.05)	.08	0.71 (0.42-1.19)	.19	0.54 (0.31-0.94)	.03	0.91 (0.47-1.76)	.77
Prequit time		0.99 (0.97-1.02)	.51	0.97 (0.94-1.00)	.08	1.01 (0.98-1.05)	.50	0.99 (0.95-1.03)	.66

^a^Number of users who had not dropped out of the intervention and had the opportunity to respond (or not) each time smoking status was assessed.

^b^aOR: adjusted odds ratio.

^c^AI/AN: American Indian/Alaska Native.

^d^NHPI: Native Hawaiian and Pacific Islander.

**Table 6:** Associations between user characteristics and response rates: from days 28-72, imputed data.

Characteristics		Day 28 (n=642)^a^		Day 35 (n=606)^a^		Day 42 (n=578)^a^		Day 72 (n=535)^a^	
		aOR^b^ (95% CI)	*P* value	aOR (95% CI)	*P* value	aOR (95% CI)	*P* value	aOR (95% CI)	*P* value
Age (winsorized)		0.99 (0.96-1.03)	.65	1.01 (0.97-1.05)	.65	1.01 (0.97-1.05)	.78	0.98 (0.93-1.04)	.58
**Race and ethnicity (reference: white)**									
	Black	1.15 (0.62-2.16)	.65	0.36 (0.15-0.91)	.03	0.67 (0.30-1.49)	.32	0.86 (0.29-2.52)	.78
	Latina	0.63 (0.21-1.89)	.41	0.53 (0.15-1.83)	.31	0.93 (0.30-2.89)	.90	1.03 (0.27-3.90)	.96
	Multiracial, Asian, AI/AN^c^, NHPI^d^, other	0.94 (0.41-2.17)	.89	0.79 (0.31-2.02)	.63	1.34 (0.54-3.32)	.52	1.38 (0.46-4.13)	.56
**Education (reference: college degree or higher)**									
	High school or less	0.55 (0.22-1.40)	.21	0.73 (0.26-2.07)	.56	0.70 (0.26-1.94)	.50	0.59 (0.15-2.38)	.46
	Some college	1.09 (0.49-2.44)	.83	1.48 (0.57-3.83)	.42	1.39 (0.54-3.54)	.49	0.92 (0.27-3.19)	.90
**Region (reference: South)**									
	Northeast	1.16 (0.58-2.34)	.68	0.84 (0.36-1.97)	.69	1.19 (0.52-2.74)	.68	0.79 (0.24-2.58)	.70
	Midwest	0.66 (0.34-1.26)	.21	0.89 (0.45-1.75)	.73	1.44 (0.75-2.77)	.27	0.81 (0.30-2.16)	.67
	West	0.82 (0.39-1.71)	.60	0.85 (0.38-1.91)	.69	0.39 (0.13-1.18)	.10	1.26 (0.47-3.36)	.64
**Cigarettes per day (reference: light)**									
	Moderate	1.09 (0.63-1.90)	.75	1.50 (0.83-2.71)	.18	0.89 (0.46-1.70)	.72	1.90 (0.83-4.33)	.13
	Heavy	0.78 (0.22-2.79)	.71	0.60 (0.13-2.75)	.51	0.86 (0.24-3.14)	.82	0.84 (0.10-6.96)	.87
**Smoking frequency (reference: nondaily)**									
	Daily	0.60 (0.31-1.17)	.14	0.62 (0.29-1.34)	.23	0.72 (0.32-1.60)	.42	0.36 (0.15-0.91)	.03
Prequit time		0.99 (0.94-1.03)	.54	1.00 (0.95-1.05)	.92	1.01 (0.96-1.06)	.73	0.95 (0.88-1.01)	.10

^a^Number of users who had not dropped out of the intervention and had the opportunity to respond (or not) each time smoking status was assessed; on day 72, in addition to dropouts on quit day through day 42, we excluded users whose quit days were 43-71 days before the end of the study and those who opted out on days 43-71 because they did not receive the day 72 prompt.

^b^aOR: adjusted odds ratio.

^c^AI/AN: American Indian/Alaska Native.

^d^NHPI: Native Hawaiian and Pacific Islander.

**Table 7:** Associations between user characteristics and abstinence rates: from quit date to days 7-21, imputed data.

Characteristics		Quit date (n=1082)^a^		Day 7 (n=1082)^a^		Day 14 (n=1082)^a^		Day 21 (n=1082)^a^	
		aOR^b^ (95% CI)	*P* value	aOR (95% CI)	*P* value	aOR (95% CI)	*P* value	aOR (95% CI)	*P* value
Age (winsorized)		1.01 (0.99-1.04)	.34	1.00 (0.96-1.04)	.88	1.01 (0.97-1.05)	.63	1.00 (0.95-1.04)	.95
**Race and ethnicity (reference: white)**									
	Black	1.27 (0.78-2.05)	.34	1.11 (0.55-2.22)	.77	1.16 (0.59-2.29)	.67	1.32 (0.64-2.70)	.45
	Latina	0.72 (0.35-1.47)	.37	0.78 (0.29-2.09)	.62	0.98 (0.36-2.62)	.96	0.22 (0.03-1.62)	.14
	Multiracial, Asian, AI/AN^c^, NHPI^d^, other	0.97 (0.52-1.80)	.92	1.26 (0.58-2.74)	.57	1.01 (0.41-2.50)	.99	0.80 (0.27-2.34)	.68
**Education (reference: college degree or higher)**									
	High school or less	0.59 (0.33-1.06)	.08	0.39 (0.17-0.92)	.03	0.57 (0.23-1.38)	.21	0.87 (0.24-3.14)	.84
	Some college	0.94 (0.56-1.57)	.80	0.82 (0.40-1.65)	.57	1.05 (0.49-2.27)	.90	1.41 (0.48-4.15)	.53
**Region (reference: South)**									
	Northeast	0.83 (0.48-1.46)	.52	0.76 (0.35-1.63)	.47	0.66 (0.29-1.47)	.31	1.10 (0.50-2.43)	.81
	Midwest	0.99 (0.64-1.53)	.96	0.42 (0.20-0.88)	.02	0.75 (0.40-1.41)	.37	0.73 (0.36-1.47)	.38
	West	1.31 (0.81-2.14)	.27	1.10 (0.57-2.13)	.77	0.60 (0.27-1.36)	.22	0.42 (0.14-1.24)	.12
**Cigarettes per day (reference: light)**									
	Moderate	1.14 (0.76-1.71)	.53	0.93 (0.52-1.66)	.80	1.17 (0.65-2.11)	.61	1.02 (0.54-1.91)	.96
	Heavy	1.06 (0.51-2.21)	.87	0.20 (0.03-1.49)	.12	0.75 (0.22-2.60)	.66	0.52 (0.12-2.27)	.38
**Smoking frequency (reference: nondaily)**									
	Daily	0.42 (0.26-0.67)	<.001	0.60 (0.31-1.18)	.14	0.38 (0.20-0.74)	.004	1.23 (0.46-3.25)	.68
Prequit time		0.96 (0.93-0.99)	.02	0.96 (0.92-1.01)	.08	0.99 (0.95-1.04)	.73	0.98 (0.94-1.03)	.50

^a^N=1082 reflects total users who made it to quit date.

^b^aOR: adjusted odds ratio.

^c^AI/AN: American Indian/Alaska Native.

^d^NHPI: Native Hawaiian and Pacific Islander.

**Table 8:** Associations between user characteristics and abstinence rates: from days 28-72, imputed data.

Characteristics		Day 28 (n=1082)^a^		Day 35 (n=1082)^a^		Day 42 (n=1082)^a^		Day 72 (n=1053)^a^	
		aOR^b^ (95% CI)	*P* value	aOR (95% CI)	*P* value	aOR (95% CI)	*P* value	aOR (95% CI)	*P* value
Age (winsorized)		1.00 (0.96-1.05)	.94	1.02 (0.97-1.07)	.53	0.99 (0.94-1.04)	.70	1.00 (0.93-1.07)	.90
**Race and ethnicity (reference: white)**									
	Black	1.71 (0.85-3.42)	.13	0.68 (0.25-1.90)	.47	0.85 (0.32-2.25)	.75	0.76 (0.19-2.99)	.70
	Latina	0.95 (0.32-2.83)	.93	0.68 (0.15-3.00)	.61	0.78 (0.17-3.44)	.74	1.20 (0.25-5.73)	.82
	Multiracial, Asian, AI/AN^c^, NHPI^d^, other	1.45 (0.61-3.47)	.40	0.62 (0.14-2.70)	.52	1.74 (0.63-4.81)	.29	1.14 (0.24-5.40)	.87
**Education (reference: college degree or higher)**									
	High school or less	0.59 (0.20-1.74)	.34	0.67 (0.19-2.34)	.53	0.93 (0.27-3.13)	.90	1.27 (0.18-9.22)	.81
	Some college	1.28 (0.52-3.15)	.59	1.30 (0.45-3.72)	.62	1.51 (0.51-4.50)	.46	1.80 (0.25-12.71)	.55
**Region (reference: South)**									
	Northeast	0.68 (0.29-1.61)	.38	0.51 (0.17-1.54)	.23	0.60 (0.20-1.80)	.36	Undefined^e^	N/A^f^
	Midwest	0.63 (0.31-1.28)	.20)	0.83 (0.39-1.76)	.62	0.94 (0.44-1.99)	.86	0.48 (0.15-1.49)	.20
	West	0.82 (0.37-1.80)	.61	0.26 (0.06-1.12)	.07	0.24 (0.05-1.04)	.06	0.57 (0.16-2.11)	.40
**Cigarettes per day (reference: light)**									
	Moderate	1.15 (0.62-2.15)	.66	2.31 (1.12-4.75)	.02	1.40 (0.66-2.96)	.38	2.29 (0.82-6.44)	.12
	Heavy	0.59 (0.13-2.58)	.48	1.00 (0.21-4.64)	>.99	0.91 (0.20-4.14)	.90	1.01 (0.12-8.63)	.99
**Smoking frequency (reference: nondaily)**									
	Daily	0.54 (0.26-1.12)	.10	0.58 (0.21-1.62)	.30	0.51 (0.20-1.34)	.17	0.27 (0.08-0.89)	.03
Prequit time		0.98 (0.93-1.03)	.37	1.00 (0.94-1.06)	>.99	1.00 (0.95-1.06)	.94	0.94 (0.87-1.03)	.17

^a^N=1082 reflects total users who made it to quit date. On day 72, n=1053 because 29 users had quit 43-71 days before the end of the study and did not have the opportunity to respond to the day 72 smoking status prompt.

^b^aOR: adjusted odds ratio.

^c^AI/AN: American Indian/Alaska Native.

^d^NHPI: Native Hawaiian and Pacific Islander.

^e^Due to quasi-complete separation of data points, aOR was undefined.

^f^N/A: not applicable.

## Discussion

### Principal Findings

In a real-world implementation of SmokefreeMOM, an SMS text messaging smoking cessation intervention targeting pregnant women, racial and ethnic composition of users did not mirror the national rates of smoking during pregnancy. Users dropped out as early as the sign-up date and continued to drop out throughout the prequit and intervention periods, with fewer than half completing the intervention. Black women, users with some college education, and users with a high school education or less had a lower likelihood of dropping out of SmokefreeMOM. Nonetheless, response and abstinence rates did not differ by race and ethnicity or by education at key milestones: quit day, intervention end, and 1-month follow-up. Efforts are needed to ensure that SmokefreeMOM reaches and engages pregnant smokers and helps them achieve smoking abstinence, particularly minorities and those with lower educational attainment with comparatively high rates of smoking during pregnancy.

The demographic composition of SmokefreeMOM users revealed adequate enrollment of pregnant smokers of low educational attainment but inadequate enrollment of racial and ethnic minorities. Specifically, women with some college education or high school education or less, who have high rates of smoking during pregnancy (8%-12%) [33], represented 82.68% (1065/1288) of SmokefreeMOM users by educational attainment in our sample. In contrast, AI/AN women, who have the highest rates of smoking during pregnancy by race and ethnicity, as well as NHPI, Asian, and multiracial women, were underrepresented, with AI/AN (9/1230), NHPI (6/1230), and Asian (14/1230) women representing 2.35% and multiracial women representing 4.72% (58/1230) of users. Efforts to overenroll marginalized populations is paramount given their limited access to smoking cessation resources and high risk of exacerbated smoking-related health problems.

Efforts should be directed to improve SmokefreeMOM’s overall reach and early uptake among women who intend to become pregnant. Over 4 years, SmokefreeMOM had 2764 user records, meaning that SmokefreeMOM reached less than 1% of roughly a million pregnant smokers (250,000 pregnant smokers per year) [4]. Aside from *how many* women SmokefreeMOM reached, *when* it reached them is equally important. On average, SmokefreeMOM users were in their second trimester, with 5 months until their due dates. A potential approach is to integrate SmokefreeMOM in clinical care for women of reproductive age, as is done with text4baby, an SMS text messaging intervention targeting pregnant women and new mothers with tailored messages about their pregnancy or their babies’ development [34]. This integration should include Planned Parenthood and federally qualified health centers to reach underserved women to whom enrolling in SmokefreeMOM solely or in combination with other cessation aids can be recommended [35].

SmokefreeMOM produced somewhat poorer abstinence rates than other smoking cessation interventions. Among SmokefreeMOM users, 1.99% (21/1053) were abstinent at 1 month postintervention. Other SMS text messaging interventions for pregnant women showed 7% to 20% abstinence rates at 1-month follow-up [20,36]. These were incentivized, randomized trials, which may account for the higher abstinence rates. However, abstinence rates among users of SmokefreeTXT, the National Cancer Institute’s publicly available SMS text messaging smoking cessation intervention for the general population, were also higher, at 7.2% [37]. Notably, nonresponse for smoking status questions was high and, to be conservative, we considered nonresponders to be smokers. However, nonresponse could signify passive disengagement from the intervention wherein some users failed to text “STOP” to drop out. Conversely, nonresponders or dropouts could be those who quit smoking and had disengaged from messages to avoid smoking relapse triggers [20]. Indeed, 5 of 509 users reported that they were abstinent on the day they dropped out. Increasing responses rates is necessary to accurately estimate abstinence outcomes of SmokefreeMOM.

The lower dropout rates among black users and those with some college education or with high school education or less may be attributed to a greater motivation to quit smoking or lack of access to alternative cessation aids [38]. Conversely, response and abstinence rates were uniform across all user characteristics. Thus, lower dropout among black users and those with less than college education did not translate to better response and abstinence rates. Although program dose has been predictive of abstinence in other SMS text messaging interventions [16], this association varies by many factors (eg, length of program, number of messages received). For example, abstainers in Text2Quit were enrolled longer than nonabstainers, but an overall program dose measure was not associated with smoking abstinence [39]. Lower abstinence rates are consistent with prior research that finds that black and less-educated smokers struggle with smoking abstinence more than white and college-educated smokers do [40].

The need to improve user retention, interaction with SmokefreeMOM until intervention end, and abstinence rates points to engagement as a coveted strategy [41,42]. Engagement strategies could involve incorporating additional keywords the user can text to interact with the intervention or providing congratulatory messages for interaction [43]. Importantly, the best strategy may sometimes be to temporarily provide nothing to the user. At times, not responding to a prompt can signal intervention fatigue, and providing additional content could have negative impacts on user engagement and progress [44]. SMS text messaging interventions must be balanced to engage users but avoid overwhelming them at the same time. Indeed, SMS text messaging interventions that decrease or allow personalized adjustment of message frequency are more effective than those with a fixed frequency [45].

Research is needed to increase SmokefreeMOM’s effectiveness, particularly among high-need minorities and marginalized populations. Of interest is fully utilizing mobile technology to deploy race- and ethnicity-specific message libraries that uniquely appeal to their respective target audiences. These types of targeted programs are desired by racial and ethnicity minority populations [18] and are likely to increase engagement and smoking abstinence [46]. Importantly, SMS text messaging interventions can still be inaccessible to low-income women, due to the cost of receiving or sending messages under limited SMS text messaging plans. Accordingly, providing SmokefreeMOM users with credit toward cell phone bills or making text messages to and from intervention services free of charge, currently done by text4baby [34,47], may be a necessary investment. Such proposals are foreseeable given that most health insurance plans cover smoking treatments to varying degrees [48].

Consistent with previous studies [37,41], in this study, daily smoking was a risk factor for poor response and abstinence rates. Longer prequit time was associated with a lower likelihood of dropping out but a lower likelihood of abstinence at quit day. To address the unique needs of daily smokers attempting to quit, a stepped-up approach that incorporates supplemental cessation aids or a tailored message library in its content and delivery schedule, or both, may be needed [49,50]. More information is needed to understand the benefits and downsides to incorporating a prequit period among pregnant women. Although evidence suggests that a planning period prior to quitting is associated with a higher likelihood of abstinence [26,51], this research focused on the general population of smokers. However, pregnant women are more likely to spontaneously quit than other smokers [52] and, thus, their quit attempts may be more successful if implemented immediately.

### Strengths and Limitations

This study reflects the strengths and limitations of the real-world implementation of public health interventions. Real-world observational data are invaluable to assess the effectiveness, generalizability, and implementation fidelity of evidence-based interventions in real settings and to inform future experimental studies and trials in a time- and cost-efficient way [53]. SmokefreeMOM users are heterogeneous compared with those enrolled in studies with stringent inclusion criteria, such as randomized controlled trials. Users self-selected to enroll, wanted to quit smoking, and owned a mobile device, presumably with SMS text messaging plans that allowed receipt of multiple messages a day. This study did not capture technical issues common in SMS text messaging interventions [20] that could have affected study outcomes. We observed high levels of missing data, low response rates (especially as the intervention progressed), and high dropout rates, likely due to a lack of the monetary compensation that is typical of researcher-controlled studies. Low response rates beyond the 1-month follow-up prevented us from assessing postnatal relapse, although as many as 50% of women return to smoking after pregnancy [5], placing their babies at risk of secondhand smoke [6].

We operationalized dropout to include SmokefreeMOM users who texted “STOP” (ie, active dropout) or were unreachable, which could have inflated our dropout rates if unreachable women had reenrolled with a new phone number or had cell phone service stopped due to financial constraints. This operationalization did not capture passive dropouts, that is, users who did not respond to sequential smoking status prompts. Disentangling active and passive dropouts in future work many inform the development and implementation of tailored engagement strategies. Although response and abstinence rates were uniform across racial and ethnic groups in this study, adequate sample sizes could unveil differences in response and abstinence rates previously documented [54]. Future studies can incentivize enrollment and retention to allow for adequate power to assess program outcomes across racial and ethnic groups and socioeconomic gradients.

### Conclusions

SMS text messaging interventions are efficacious for smoking cessation [16,45]. SmokefreeMOM, a freely available cessation intervention, is a necessary resource for a hard-to-reach population of pregnant smokers, especially underserved racial and ethnic minorities. Overall abstinence rates among SmokefreeMOM users were lower than among other smoking cessation SMS text messaging interventions. Response and abstinence rates were equivalent across all demographic characteristics of SmokefreeMOM users. Black and less-educated women were more likely to remain in the intervention until its end, presenting opportunities to enhance their engagement and, subsequently, abstinence rates. Research into strategies to increase the reach, engagement, and effectiveness of SmokefreeMOM, particularly for racial and ethnic minority and other marginalized populations with high rates of smoking during pregnancy, is critical for reducing smoking among pregnant women across the United States.

## Appendix

Multimedia Appendix 1 SmokefreeMOM user characteristics, complete case data.

Multimedia Appendix 2 Association between user characteristics and dropout.

Multimedia Appendix 3 Response and abstinence rates by race and ethnicity, imputed data.

Multimedia Appendix 4 Associations between user characteristics and responses rates and abstinence, complete case analysis.

## Abbreviations

AI/AN: American Indian/Alaska Native
aOR: adjusted odds ratio
HR: hazard ratio
IQR: interquartile range
NHPI: Native Hawaiian and Pacific Islander
SMS: short message service
