# Supplementary material for: Dropout and Abstinence Outcomes in a National Text Messaging Smoking Cessation Intervention for Pregnant Women, SmokefreeMOM: Observational Study
Source: JMIR Mhealth Uhealth. 2019 Oct 7;7(10):e14699. doi: 10.2196/14699 (PMC6803886; doi:10.2196/14699)
Supplement: Multimedia Appendix 1 [file mhealth_v7i10e14699_app1.pdf]

Multimedia Appendix 1. SFMom user characteristics, complete case data.

	Total		Non-completers[a]		Completers[b]	
	N	Summary Statistic	N	Summary Statistic	N	Summary Statistic
**Age**						
Mean (SD)	1288	29.58 (7.64)	715	29.64 (7.68)	573	29.49 (7.60)
5% Trimmed Mean (SD)	1158	29.02 (6.35)	643	29.09 (6.33)	515	28.93 (6.38)
Range	1288	14 - 99	715	16 - 99	573	14 - 72
Median (IQR)	1288	29.00 (10.00)	715	29.00 (10.00)	573	28.00 (10.00)
**Age (Winsorized)**						
Mean (SD)	1288	29.46 (7.11)	715	29.52 (7.05)	573	29.39 (7.19)
5% Trimmed Mean (SD)	1158	29.02 (6.35)	643	29.09 (6.33)	515	28.93 (6.38)
Range	1288	14 - 54	715	16 - 54	573	14 - 54
Median (IQR)	1288	29.00 (10.00)	715	29.00 (10.00)	573	28.00 (10.00)
**Race/Ethnicity, %**						
White	817	63.43	484	67.69	333	58.12
Black	191	14.83	78	10.91	113	19.72
Latina	109	8.46	66	9.23	43	7.50
Multiracial, Asian, AI/AN, NHPI, Other[c]	113	8.77	56	7.83	57	9.95
Missing	58	4.50	31	4.34	27	4.71
**Education, %**						
High school or less	385	29.89	201	28.11	184	32.11
Some college	415	32.22	231	32.31	184	32.11
College graduate or above	168	13.04	117	16.36	51	8.90
Missing	320	24.84	166	23.22	154	26.88
**Region,[d] %**						
Northeast	178	13.82	105	14.69	73	12.74
Midwest	329	25.54	200	27.97	129	22.51
South	565	43.87	293	40.98	272	47.47
West	206	15.99	116	16.22	90	15.71
Missing	10	0.78	1	0.14	9	1.57
**Smoking frequency, %**						
Non-daily	141	10.95	66	9.23	75	13.09
Daily	1130	87.73	639	89.37	491	85.69
Missing	17	1.32	10	1.40	7	1.22
**Cigarettes per day, %**						
Light (<10 cigarettes)	756	58.70	394	55.10	362	63.18
Moderate (11-19 cigarettes)	412	31.99	238	33.29	174	30.37
Heavy (≥20 cigarettes)	110	8.54	77	10.77	33	5.76
Missing	10	0.78	6	0.84	4	0.70
**Web-enabled phone, %**						
Yes	1238	96.12	687	96.08	551	96.16
No	50	3.88	28	3.92	22	3.84

**Time of dropout,[e] %**						
Prior to quit date	206	15.99	206	28.81	N/A	N/A
On or after quit date	509	39.52	509	71.19	N/A	N/A
**Time from signup date to due date[f]**						
Mean (SD)	1288	161.21 (73.65)	715	164.73 (75.57)	573	156.82 (71.01)
5% Trimmed Mean (SD)	1158	164.49 (75.93)	643	168.45 (78.25)	515	159.47 (72.34)
Range	1288	0 - 279	715	0 - 279	573	0 - 279
Median (IQR)	1288	175.50 (117.00)	715	184.00 (113.00)	573	167.00 (113.00)
**Time from signup to quit date[f]**						
Mean (SD)	1288	10.41 (15.74)	715	9.38 (14.06)	573	11.70 (17.53)
5% Trimmed Mean (SD)	1158	8.04 (9.60)	643	7.58 (7.65)	515	8.93 (12.65)
Range	1288	0 - 197	715	0 - 197	573	0 - 144
Median (IQR)	1288	7.00 (13.00)	715	7.00 (13.00)	573	7.00 (13.00)
**Prequit time[f]**						
Mean (SD)	1288	6.12 (5.53)	715	5.20 (5.29)	573	7.28 (5.61)
5% Trimmed Mean (SD)	1158	6.03 (5.83)	643	5.00 (5.58)	515	7.31 (5.91)
Range	1288	0 - 14	715	0 - 14	573	0 - 14
Median (IQR)	1288	5.00 (12.00)	715	3.00 (10.00)	573	7.00 (13.00)
**Time from quit day to dropout[f,g]**						
Mean (SD)	509	10.70 (11.74)	509	10.70 (11.74)	N/A	N/A
5% Trimmed Mean (SD)	457	9.67 (11.92)	457	9.67 (11.92)	N/A	N/A
Range	509	0 - 42	509	0 - 42	N/A	N/A
Median (IQR)	509	6.00 (16.00)	509	6.00 (16.00)	N/A	N/A

[a]Non-completers are users who opted out of the intervention any time on or between day -14 (14 days before quit date) and intervention end (day 42)

[b]Completers are users who remained in SFMom until after intervention end

[c]Complete case sample was 4.72% (58/1230) multiracial, 1.14% (14/1230) Asian, 0.73% (9/1230) AI/AN, 0.49% (6/1230) NHPI, and 2.11% (26/1230) other

[d]Users provided their zip codes, which were automatically converted into US state. We categorized states into US Census Bureau region. One user who lived in Puerto Rico, for which there is no census region, was categorized into South to retain her data in analyses

[e]Remaining 44.49% (573/1288) did not drop out prior to intervention end

[f]All time variables are reported in number of days

[g]Among those who made it to their quit date
