# Supplementary material for: Dropout and Abstinence Outcomes in a National Text Messaging Smoking Cessation Intervention for Pregnant Women, SmokefreeMOM: Observational Study
Source: JMIR Mhealth Uhealth. 2019 Oct 7;7(10):e14699. doi: 10.2196/14699 (PMC6803886; doi:10.2196/14699)
Supplement: Multimedia Appendix 2 [file mhealth_v7i10e14699_app2.pdf]

Multimedia Appendix 2. Association between user characteristics and dropout (*n* = 1288)

Characteristic	Adjusted Odds Ratio	95% CI	*p*-value
Age (Winsorized)	0.99	0.97 - 1.01	.29
**Race/Ethnicity** (ref: White)			
Black	0.63	0.45 - 0.88	.007
Latina	0.91	0.60 - 1.40	.68
Multiracial, Asian, AI/AN, NHPI, other	0.69	0.46 - 1.03	.07
**Education** (ref: ≥College graduate)			
≤High school	0.53	0.35 - 0.81	.003
Some college	0.62	0.42 - 0.91	.02
**Region** (ref: South)			
Northeast	1.25	0.87 - 1.78	.23
Midwest	1.34	1.00 - 1.78	.048
West	1.13	0.81 - 1.60	.47
**Cigarettes per day** (ref: Light)			
Moderate	1.11	0.85 - 1.45	.44
Heavy	1.83	1.16 - 2.89	.01
**Smoking frequency** (ref: Non-daily)			
Daily	1.41	0.97 - 2.06	.07
Prequit time	0.93	0.91 - 0.95	<.001

Note: Users who dropped out in the prequit period, on quit day, or on/before day 42 were considered dropouts in this analysis. All those who remained in the program past day 42 were considered to have completed the program.
