# Supplementary material for: Dropout and Abstinence Outcomes in a National Text Messaging Smoking Cessation Intervention for Pregnant Women, SmokefreeMOM: Observational Study
Source: JMIR Mhealth Uhealth. 2019 Oct 7;7(10):e14699. doi: 10.2196/14699 (PMC6803886; doi:10.2196/14699)
Supplement: Multimedia Appendix 3 [file mhealth_v7i10e14699_app3.pdf]

Multimedia Appendix 3. Response and abstinence rates by race/ethnicity, imputed data.

| | Total[a] | Abstinent | Not Abstinent | No Response | Response Rate[b] | Abstinence Point Prevalence[c] |
|---|---|---|---|---|---|---|
| | ***N*** | ***N*** | ***N*** | ***N*** | **%** | **%** |
| **Quit Date** | | | | | | |
| Overall | 1,082 | 157 | 162 | 763 | 29.48 | 14.51 |
| White | 709 | 101 | 118 | 491 | 30.76 | 14.18 |
| Black | 181 | 31 | 23 | 127 | 29.97 | 17.15 |
| Latina | 91 | 11 | 8 | 73 | 20.41 | 11.57 |
| Multiracial, Asian, AI/AN, NHPI, Other | 100 | 15 | 13 | 72 | 27.85 | 14.75 |
| **7-days post-quit** | | | | | | |
| Overall | 813 | 70 | 80 | 663 | 18.45 | 6.47 |
| White | 519 | 43 | 60 | 416 | 19.81 | 6.04 |
| Black | 151 | 13 | 10 | 128 | 15.15 | 7.17 |
| Latina | 60 | 5 | 3 | 52 | 13.88 | 5.65 |
| Multiracial, Asian, AI/AN, NHPI, Other | 83 | 9 | 7 | 67 | 19.24 | 8.97 |
| **14-days post-quit** | | | | | | |
| Overall | 727 | 67 | 65 | 595 | 18.16 | 6.19 |
| White | 453 | 42 | 41 | 371 | 18.21 | 5.92 |
| Black | 144 | 14 | 7 | 123 | 14.73 | 7.72 |
| Latina | 55 | 5 | 5 | 44 | 18.68 | 5.54 |
| Multiracial, Asian, AI/AN, NHPI, Other | 75 | 6 | 12 | 57 | 24.02 | 5.98 |
| **21-days post-quit** | | | | | | |
| Overall | 678 | 54 | 51 | 573 | 15.49 | 4.99 |
| White | 421 | 36 | 34 | 351 | 16.60 | 5.08 |
| Black | 136 | 13 | 9 | 114 | 16.16 | 7.17 |
| Latina | 52 | 1 | 2 | 49 | 5.83 | 1.10 |
| Multiracial, Asian, AI/AN, NHPI, Other | 68 | 4 | 6 | 58 | 14.67 | 3.99 |
| **28-days post-quit** | | | | | | |
| Overall | 642 | 59 | 20 | 563 | 12.31 | 5.45 |

| | | | | | | |
|---|---|---|---|---|---|---|
| White | 396 | 33 | 16 | 347 | 12.35 | 4.65 |
| Black | 131 | 15 | 3 | 113 | 13.73 | 8.27 |
| Latina | 49 | 4 | 0 | 45 | 8.21 | 4.39 |
| Multiracial, Asian, AI/AN, NHPI, Other | 65 | 7 | 1 | 57 | 12.28 | 6.98 |
| **35-days post-quit** | | | | | | |
| Overall | 606 | 39 | 23 | 544 | 10.23 | 3.60 |
| White | 370 | 30 | 17 | 323 | 12.69 | 4.23 |
| Black | 125 | 5 | 1 | 119 | 4.80 | 2.76 |
| Latina | 48 | 2 | 1 | 45 | 6.20 | 2.19 |
| Multiracial, Asian, AI/AN, NHPI, Other | 62 | 2 | 4 | 56 | 9.65 | 1.99 |
| **42-days post-quit** | | | | | | |
| Overall | 578 | 38 | 19 | 521 | 9.86 | 3.51 |
| White | 353 | 25 | 12 | 316 | 10.49 | 3.53 |
| Black | 119 | 6 | 3 | 110 | 7.56 | 3.31 |
| Latina | 46 | 2 | 2 | 42 | 8.64 | 2.19 |
| Multiracial, Asian, AI/AN, NHPI, Other | 60 | 5 | 2 | 53 | 11.66 | 4.98 |
| **72-days post-quit** | | | | | | |
| Overall | 535 | 21 | 12 | 502 | 6.17 | 1.99 |
| White | 327 | 14 | 6 | 307 | 6.12 | 2.02 |
| Black | 108 | 3 | 2 | 103 | 4.63 | 1.73 |
| Latina | 44 | 2 | 1 | 41 | 6.83 | 2.30 |
| Multiracial, Asian, AI/AN, NHPI, Other | 56 | 2 | 3 | 51 | 8.87 | 2.04 |

[a]Total *N* reflects imputed data and is 1/20th of a subject rounded to the nearest integer.
[b]Response rate = ((*N* Abstinent + *N* Not Abstinent)/Total *N*) x 100
[c]Point prevalence = (Abstinent /*N* for point prevalence) x 100. N for point prevalence calculations from quit day to day 42 are: 1,082 (overall), 709 (White), 181 (Black), 91 (Latina), 100 (Multiracial, Asian, AI/AN, NHPI, Other); *N* for point prevalence calculations at day 72 are: 1,053 (overall), 694 (White), 173 (Black), 87 (Latina), 98 (Multiracial, Asian, AI/AN, NHPI, Other)
