# Supplementary material for: Dropout and Abstinence Outcomes in a National Text Messaging Smoking Cessation Intervention for Pregnant Women, SmokefreeMOM: Observational Study
Source: JMIR Mhealth Uhealth. 2019 Oct 7;7(10):e14699. doi: 10.2196/14699 (PMC6803886; doi:10.2196/14699)
Supplement: Multimedia Appendix 4 [file mhealth_v7i10e14699_app4.pdf]

Multimedia Appendix 4. Associations between user characteristics and response and abstinence rates, complete case analysis.

	Response Rate[a]			
	**Quit Date** ***n* = 753**	**Day 7** ***n* = 551**	**Day 14** ***n* = 496**	**Day 21** ***n* = 463**
Characteristic	aOR (95% CI) (*p*-value)	aOR (95% CI) (*p*-value)	aOR (95% CI) (*p*-value)	aOR (95% CI) (*p*-value)
Age (Winsorized)	0.98 (0.96-1.01) (.20)	0.97 (0.93-1.01) (.14)	0.99 (0.95-1.03) (.64)	1.01 (0.97-1.05) (.56)
**Race/Ethnicity** (Ref: White)				
Black	0.89 (0.56-1.42) (.62)	0.74 (0.39-1.44) (.38)	0.65 (0.31-1.34) (.24)	1.12 (0.56-2.22) (.75)
Latina	0.40 (0.19-0.86) (.02)	0.40 (0.13-1.24) (.11)	1.35 (0.49-3.68) (.56)	0.36 (0.08-1.67) (.19)
Multiracial, Asian, AI/AN, NHPI, Other	0.83 (0.45-1.53) (.56)	0.75 (0.34-1.67) (.48)	0.89 (0.36-2.20) (.81)	0.82 (0.29-2.33) (.71)
**Education** (Ref: ≥college degree)				
≤High school	0.52 (0.32-0.85) (.01)	0.18 (0.09-0.36) (<.001)	0.40 (0.18-0.88) (.02)	0.63 (0.24-1.66) (.35)
Some college	0.93 (0.59-1.47) (.76)	0.54 (0.30-0.95) (.03)	1.19 (0.59-2.38) (.62)	1.69 (0.72-3.98) (.23)
**Region** (ref: South)				
Northeast	1.17 (0.71-1.93) (.55)	1.89 (0.98-3.62) (.06)	1.44 (0.70-2.98) (.32)	2.00 (0.92-4.36) (.08)
Midwest	1.04 (0.70-1.55) (.84)	1.22 (0.69-2.16) (.49)	1.34 (0.72-2.50) (.36)	1.98 (1.06-3.71) (.03)
West	1.01 (0.63-1.62) (.95)	1.28 (0.67-2.47) (.45)	1.04 (0.48-2.25) (.93)	0.57 (0.20-1.60) (.28)
**Cigarettes per day** (Ref: Light)				
Moderate	1.17 (0.81-1.68) (.40)	0.88 (0.53-1.47) (.62)	1.07 (0.60-1.91) (.83)	0.60 (0.32-1.14) (.12)
Heavy	1.59 (0.81-3.12) (.18)	1.25 (0.39-4.06) (.71)	2.92 (0.99-8.61) (.052)	3.16 (0.96-10.4) (.06)
**Smoking Frequency** (Ref: Non-daily)				
Daily	0.78 (0.46-1.30) (.34)	0.84 (0.43-1.63) (.61)	0.46 (0.23-0.92) (.03)	1.37 (0.57-3.28) (.48)
Pre-quit time	1.02 (0.99-1.05) (.20)	0.99 (0.95-1.03) (.76)	1.03 (0.99-1.08) (.14)	1.01 (0.96-1.06) (.76)

Multimedia Appendix 4. Associations between user characteristics and response rates and abstinence (complete case analysis), continued

	**Day 28**	**Day 35**	**Day 42**	**Day 72**
	***n* = 437**	***n* = 409**	***n* = 392**	***n* = 361**
Characteristic	aOR (95% CI) (*p*-value)	aOR (95% CI) (*p*-value)	aOR (95% CI) (*p*-value)	aOR (95% CI) (*p*-value)
Age (Winsorized)	0.99 (0.94-1.04) (.69)	1.01 (0.96-1.06) (.74)	1.00 (0.96-1.05) (.92)	0.99 (0.91-1.07) (.74)
**Race/Ethnicity** (Ref: White)				
Black	1.11 (0.51-2.42) (.79)	0.25 (0.08-0.76) (.01)	0.55 (0.21-1.47) (.23)	0.76 (0.18-3.15) (.70)
Latina	0.58 (0.12-2.72) (.49)	0.53 (0.11-2.51) (.42)	1.18 (0.30-4.58) (.81)	0.60 (0.07-5.53) (.65)
Multiracial, Asian, AI/AN, NHPI, Other	0.85 (0.27-2.66) (.78)	0.60 (0.16-2.17) (.43)	1.09 (0.34-3.52) (.89)	1.83 (0.43-7.74) (.41)
**Education** (Ref: ≥college degree)				
≤High school	0.40 (0.14-1.13) (.08)	0.81 (0.26-2.46) (.70)	0.60 (0.20-1.86) (.38)	0.47 (0.09-2.43) (.37)
Some college	1.22 (0.50-2.99) (.66)	2.19 (0.81-5.92) (.12)	1.53 (0.56-4.16) (.41)	1.05 (0.25-4.41) (.94)
**Region** (ref: South)				
Northeast	1.18 (0.50-2.81) (.71)	1.04 (0.40-2.68) (.94)	1.16 (0.43-3.13) (.76)	1.45 (0.37-5.59) (.59)
Midwest	0.54 (0.24-1.23) (.14)	0.88 (0.40-1.94) (.75)	1.41 (0.65-3.07) (.39)	0.73 (0.18-2.90) (.65)
West	0.67 (0.25-1.78) (.42)	0.64 (0.22-1.88) (.42)	0.45 (0.12-1.64) (.22)	1.09 (0.25-4.79) (.91)
**Cigarettes per day** (Ref: Light)				
Moderate	0.75 (0.37-1.53) (.42)	1.21 (0.59-2.46) (.61)	0.90 (0.41-1.97) (.79)	1.12 (0.34-3.69) (.85)
Heavy	2.47 (0.60-10.2) (.21)	1.38 (0.27-7.12) (.70)	2.37 (0.56-9.97) (.24)	2.64 (0.27-25.8) (.40)
**Smoking Frequency** (Ref: Non-daily)				
Daily	1.02 (0.41-2.50) (.97)	0.37 (0.16-0.86) (.02)	0.54 (0.22-1.33) (.18)	0.26 (0.09-0.79) (.02)
Pre-quit time	1.02 (0.97-1.08) (.40)	1.02 (0.96-1.08) (.58)	1.03 (0.97-1.09) (.39)	0.96 (0.88-1.05) (.35)

Multimedia Appendix 4. Associations between user characteristics and response rates and abstinence (complete case analysis), continued

	**Abstinence (intent-to-treat)**[b]			
	**Quit Date**	**Day 7**	**Day 14**	**Day 21**
	***n* = 753**	***n* = 753**	***n* = 753**	***n* = 753**
Characteristic	aOR (95% CI) (*p*-value)	aOR (95% CI) (*p*-value)	aOR (95% CI) (*p*-value)	aOR (95% CI) (*p*-value)
Age (Winsorized)	1.00 (0.96-1.03) (.81)	0.98 (0.93-1.04) (.54)	1.01 (0.96-1.06) (.74)	0.99 (0.94-1.05) (.81)
**Race/Ethnicity** (Ref: White)				
Black	1.19 (0.67-2.11) (.54)	0.97 (0.41-2.30) (.95)	0.79 (0.33-1.90) (.60)	1.12 (0.44-2.83) (.81)
Latina	0.39 (0.14-1.14) (.09)	0.42 (0.09-1.85) (.25)	0.56 (0.13-2.47) (.44)	Undefined[c]
Multiracial, Asian, AI/AN, NHPI, Other	0.83 (0.37-1.85) (.64)	0.85 (0.28-2.55) (.77)	0.47 (0.11-2.05) (.31)	0.32 (0.04-2.50) (.28)
**Education** (Ref: ≥college degree)				
≤High school	0.42 (0.22-0.78) (.007)	0.23 (0.08-0.61) (.003)	0.43 (0.16-1.13) (.09)	0.87 (0.24-3.12) (.83)
Some college	0.84 (0.48-1.46) (.53)	0.71 (0.33-1.50) (.37)	0.98 (0.43-2.22) (.96)	1.55 (0.50-4.82) (.45)
**Region** (ref: South)				
Northeast	0.90 (0.46-1.74) (.75)	1.12 (0.47-2.67) (.79)	0.98 (0.39-2.46) (.97)	2.19 (0.86-5.60) (.10)
Midwest	0.92 (0.55-1.54) (.75)	0.42 (0.17-1.06) (.07)	0.77 (0.35-1.70) (.52)	0.99 (0.42-2.33) (.97)
West	0.93 (0.50-1.71) (.81)	1.18 (0.53-2.64) (.68)	0.60 (0.22-1.66) (.32)	0.20 (0.03-1.54) (.12)
**Cigarettes per day** (Ref: Light)				
Moderate	1.02 (0.63-1.64) (.95)	0.68 (0.34-1.39) (.30)	0.86 (0.41-1.81) (.69)	0.90 (0.40-1.99) (.79)
Heavy	1.78 (0.78-4.05) (.17)	0.34 (0.04-2.64) (.30)	1.39 (0.38-5.07) (.62)	1.19 (0.25-5.62) (.83)
**Smoking Frequency** (Ref: Non-daily)				
Daily	0.52 (0.29-0.95) (.03)	1.25 (0.46-3.40) (.67)	0.36 (0.16-0.82) (.01)	4.25 (0.56-32.4) (.16)
Pre-quit time	0.97 (0.93-1.01) (.10)	1.00 (0.95-1.06) (.99)	1.04 (0.99-1.10) (.14)	1.02 (0.95-1.08) (.63)

Multimedia Appendix 4. Associations between user characteristics and response rates and abstinence (complete case analysis), continued

	Day 28	Day 35	Day 42	Day 72
	***n* = 753**	***n* = 753**	***n* = 753**	***n* = 724**
Characteristic	aOR (95% CI) (*p*-value)	aOR (95% CI) (*p*-value)	aOR (95% CI) (*p*-value)	aOR (95% CI) (*p*-value)
Age (Winsorized)	0.99 (0.94-1.05) (.80)	1.01 (0.95-1.08) (.65)	0.99 (0.93-1.06) (.82)	1.02 (0.91-1.13) (.78)
**Race/Ethnicity** (Ref: White)				
Black	1.74 (0.73-4.12) (.21)	0.43 (0.12-1.59) (.21)	0.45 (0.12-1.68) (.24)	0.21 (0.02-2.03) (.18)
Latina	0.91 (0.20-4.16) (.91)	0.51 (0.07-4.04) (.53)	0.60 (0.08-4.78) (.63)	Undefined
Multiracial, Asian, AI/AN, NHPI, Other	1.14 (0.32-4.06) (.83)	0.44 (0.06-3.39) (.43)	0.97 (0.21-4.44) (.97)	1.09 (0.12-9.68) (.94)
**Education** (Ref: ≥college degree)				
≤High school	0.43 (0.13-1.43) (.17)	0.56 (0.16-1.99) (.37)	0.85 (0.23-3.13) (.81)	2.15 (0.18-25.9) (.55)
Some college	1.45 (0.55-3.84) (.45)	1.48 (0.50-4.32) (.48)	1.53 (0.47-4.97) (.48)	4.35 (0.43-44.3) (.21)
**Region** (ref: South)				
Northeast	0.79 (0.28-2.22) (.65)	0.72 (0.22-2.34) (.59)	0.61 (0.17-2.28) (.46)	Undefined
Midwest	0.52 (0.20-1.35) (.18)	0.80 (0.33-1.98) (.64)	1.01 (0.42-2.43) (.98)	0.36 (0.07-1.87) (.23)
West	0.73 (0.26-2.05) (.54)	0.17 (0.02-1.31) (.09)	0.17 (0.02-1.33) (.09)	0.32 (0.04-2.75) (.30)
**Cigarettes per day** (Ref: Light)				
Moderate	0.84 (0.37-1.94) (.69)	1.63 (0.69-3.85) (.27)	1.36 (0.56-3.32) (.50)	0.64 (0.12-3.58) (.61)
Heavy	1.16 (0.25-5.41) (.85)	1.56 (0.32-7.64) (.58)	1.42 (0.29-6.90) (.66)	1.21 (0.13-11.0) (.87)
**Smoking Frequency** (Ref: Non-daily)				
Daily	0.66 (0.25-1.74) (.40)	0.36 (0.12-1.07) (.06)	0.35 (0.12-1.04) (.06)	0.17 (0.03-0.82) (.03)
Pre-quit time	1.02 (0.95-1.08) (.63)	1.04 (0.97-1.12) (.23)	1.06 (0.98-1.13) (.13)	1.01 (0.90-1.13) (.89)

[a]Response rate *n*s reflect the number of users who had not dropped out of the intervention and had the opportunity to respond (or not) each time smoking status was assessed; On Day 72, in addition to dropouts on quit day through day 42, we users whose quit days were 43-71 days before the end of study and those who opted out on days 43-71 because they did not receive the Day 72 prompt

[b]For abstinence, *n* = 753 reflects total users who made it to quit date. On Day 72, *n* = 724 because 29 users enrolled 43-71 days before the end of the study and did not have the opportunity to respond to the Day 72 smoking status prompt.

[c]Due to quasi-complete separation of data points, aOR was undefined
